# TDP-43 as a potential biomarker for amyotrophic lateral sclerosis: a systematic review and meta-analysis

**DOI:** 10.1186/s12883-018-1091-7

**Published:** 2018-06-28

**Authors:** Vivek Majumder, Jenna M. Gregory, Marcelo A. Barria, Alison Green, Suvankar Pal

**Affiliations:** 1 Centre for Clinical Brain Sciences, University of Edinburgh, Chancellor’s Building, Edinburgh, EH16 4SB UK; 2 Euan MacDonald Centre for Motor Neurone Disease Research, University of Edinburgh, Edinburgh, UK; 3 National CJD Research and Surveillance Unit, Bryan Matthews Building, Western General Hospital, Crewe Rd, Edinburgh, EH4 2XU UK

**Keywords:** TDP-43, Amyotrophic lateral sclerosis, Biomarker, Systematic review, Meta-analysis

## Abstract

**Background:**

Frontotemporal dementia (FTD) and Amyotrophic Lateral Sclerosis (ALS) are incurable, progressive and fatal neurodegenerative diseases with patients variably affected clinically by motor, behavior, and cognitive deficits. The accumulation of an RNA-binding protein, TDP-43, is the most significant pathological finding in approximately 95% of ALS cases and 50% of FTD cases, and discovery of this common pathological signature, together with an increasing understanding of the shared genetic basis of these disorders, has led to FTD and ALS being considered as part of a single disease continuum. Given the widespread aggregation and accumulation of TDP-43 in FTD-ALS spectrum disorder, TDP-43 may have potential as a biomarker in these diseases.

**Methods:**

We therefore conducted a systematic review and meta-analysis to evaluate the diagnostic utility of TDP-43 detected in the cerebrospinal fluid (CSF) of patients with FTD-ALS spectrum disorder.

**Results:**

From seven studies, our results demonstrate that patients with ALS have a statistically significantly higher level of TDP-43 in CSF (effect size 0.64, 95% CI: 0.1–1.19, *p* = 0.02).

**Conclusions:**

These data suggest promise for the use of CSF TDP-43 as a biomarker for ALS.

## Background

Frontotemporal dementia (FTD) is an umbrella term for a spectrum of neurodegenerative disorders. These disorders primarily affect behaviour, personality, executive function and language (and may include motor impairment). It is the second most common form of dementia after Alzheimer’s disease (AD) in people under 65 years old, with the average age of onset being between 45 and 65 years old, and the peak prevalence being around age 65 to 69 [1]. Prognosis is poor with a median survival of 8–10 years and a steady rate of decline [2]. Currently, there are no curative or disease modifying treatments (DMT) available, so patients are treated symptomatically [3].

FTD also affects the motor system and is now increasingly thought to be on a syndromic spectrum with amyotrophic lateral sclerosis (ALS; [4]). ALS is a neurodegenerative disease, and has a heterogeneous presentation affecting motor function, eventually affecting the ability to speak, swallow and breathe [5]. It affects both upper and lower motor neurons, but tends to spare sensory neurons. Sporadic cases of ALS have a peak incidence at around 60 years old, whereas familial cases peak at 43–52 years old. Familial cases make up 10–20% of cases [5]. Crucially, approximately 50% of patients exhibit changes in behaviour and cognition [1, 5–7], with up to 15% developing FTD [8–10]. Median survival from disease onset is 3.5 years and from diagnosis is 2 years [8], though 5–10% of patients can survive up to 10 years or more [6]. Riluzole, a sodium channel blocker which may also have other effects through inhibition of NMDA receptor signaling, glutamate release and uptake, is the only licensed DMT for ALS, and has been demonstrated to prolong life by 3–4 months [11]. There have been recent reports suggesting efficacy of Edaravone and Masitinib as additional treatments but these drugs have yet to receive widespread licensing. Mean time to diagnosis is 15 months [8] from the onset of first symptoms (ranging from 5 to 24) and relies on clinical evaluation, supported by examination and electromyography. This long diagnostic delay reflects the diagnostic challenge faced when assessing patients with a clinically heterogeneous and progressive neurodegenerative disease. The lack of robust biomarkers and diagnostic tests to support an accurate diagnosis further compounds this diagnostic challenge. The identification of biomarkers will enable early and accurate diagnosis thus avoiding unnecessary investigations and enable rapid stratification of patients into clinical trials. There are currently no biomarkers for FTD or ALS.

A substantial monogenetic component to these diseases has recently been established. The most frequent genetic abnormality is an intronic hexanucleotide repeat expansion in the gene that codes for *C9orf72*, found to be present in the majority of cases of familial cases of FTD-ALS spectrum disorders [4, 6]. Pathological assessment of specific brain regions of these cases at post-mortem reveals characteristic immuno-histochemical staining patterns of abnormally phosphorylated TDP-43 in cytoplasmic inclusion (particularly in the motor cortex, spinal cord and the frontal and temporal lobes) and p62 accumulation (particularly in the granule cell layer of the cerebellum and in the dentate gyrus of the hippocampus). In 2006, TDP-43 was identified as the main pathological finding in most sporadic and familial cases of both ALS and FTD [12]. TDP-43 is a protein with multiple functions, but is primarily involved in alternative splicing and transcriptional regulation [6]. In FTD and ALS, TDP-43 becomes ubiquinated, hyperphosphorylated and C-terminally truncated, increasing its aggregation propensity and causing widespread neurotoxicity and cell death [1]. Given the high burden of TDP-43 accumulation in the central nervous system of the majority of patients with FTD-ALS spectrum disorder, TDP-43 has been postulated as a biomarker in this disease.

A pathological diagnosis (rather than a clinical diagnosis) of FTD is referred to as frontotemporal lobar degeneration (FTLD). Indeed, three major pathological subtypes of FTLD exist characterized by the type of pathology observed in post-mortem tissue. FTLD- tau, FTLD-FUS and FTLD-TDP. These molecular subtypes are characterized respectively by the accumulation of misfolded tau, FUS and TDP-43 intracellular inclusions [13]. Due to the increasing prevalence of DMTs targeting specific molecular mechanisms there is an increasingly emergent need for biomarkers to distinguish between these subtypes. A potential biomarker, such as CSF TDP-43 could have the potential to predict the neuropathological diagnosis and thus provide us, not only with a diagnostic biomarker, but also a biomarker to enable stratification for targeted DMT development.

The aims of this study are to systematically review all studies analysing CSF TDP-43 concentrations in FTD and ALS patients and conduct a meta-analysis to investigate whether there is a significant difference between concentrations of CSF TDP-43 in patients with FTD-ALS spectrum disorders compared to neurological and non-neurological controls. Our hypothesis is that CSF TDP-43 will be significantly increased in patients with FTD-ALS spectrum disorders compared to controls.

## Methods

### Objectives

#### Population

Clinical studies of patients with FTD-ALS spectrum disorder (ALS, FTD, and FTD-ALS).

#### Intervention

CSF TDP-43 detected by ELISA or western blot.

#### Comparison

Control patients who are 1) Neurological controls (diagnosed with non-dementia neurological conditions), or 2) Non-neurological controls (diagnosed with a non-neurological condition or healthy).

#### Outcome measure

Primary outcome: effect size (concentration of TDP-43 in CSF of in FTD-ALS, FTD and ALS patients compared to controls). Secondary outcomes: Assessment of quality and heterogeneity between studies.

#### Study design

All study types where CSF TDP-43 concentrations were measured and compared to a control.

### Search methods

#### Sources

Databases: 1. PubMed, 2. Medline, 3. EMBASE 4. LILACs 5. IMEMR 6. WPRIM 7. Chinese Science Citation Index.

The date of searches was 01/03/17 and there were no publication date restrictions and no language restrictions.

#### Search terms

(Frontotemporal Lobe Dementia [MeSH] OR Amyotrophic Lateral Sclerosis [MeSH] OR Motor Neuron Disease [MeSH]) AND (Cerebrospinal Fluid [MeSH] OR Lumber Puncture [MeSH]) AND TDP [MeSH]

### Screening

The title and abstract of each paper were screened for relevance with respect to inclusion and exclusion criteria. For studies meeting our predefined criteria the full text was retrieved, imported to Excel and duplicate records were discarded. The quality of each paper was then evaluated by the QUADAS-2 quality score and relevant data were extracted.

### Eligibility

Inclusion criteria

- All studies that measure TDP-43 in the CSF of living patients with ALS or FTD, compared to controls.

Exclusion criteria

- No control group
- Animal studies
- Post mortem studies
- Reviews
- Letters and comments
- Abstracts
- Non-parametric results (Meta-Analysis only)

Study characteristics to be extracted:

- Study ID: (i) Author and (ii) Year
- Intervention: FTD (behavioural, non-fluent or semantic), ALS or both (FTD-ALS)
- Medical diagnosis of comparison/controls
- Intervention quantification method (western blot or ELISA)
- Age: mean age of patients and controls
- Sex: of patients and controls
- Diagnosis method of patients
- Genetic factors
- Sample size: of patients and controls

### Further searches

Two similar searches were done, with the similar objectives. These were to look at the effect size of lymphocytic bound TDP-43 in FTD, ALS and FTD-ALS patients compared to controls, and the effect size of plasma TDP-43 in FTD, ALS and FTD-ALS compared to controls. However, neither of these searches produced enough papers in order to do a systematic review.

#### Search terms

(Frontotemporal Lobe Dementia [MeSH] OR Amyotrophic Lateral Sclerosis [MeSH] OR Motor Neuron Disease [MeSH]) AND (Lymphocyte [MeSH] OR White Cell [MeSH] or White Blood Cell [MeSH]) AND TDP [MeSH].

(Frontotemporal Lobe Dementia [MeSH] OR Amyotrophic Lateral Sclerosis [MeSH] OR Motor Neuron Disease [MeSH]) AND (Plasma [MeSH] OR Serum [MeSH] OR Blood [MeSH]) AND TDP [MeSH]

### Statistical analysis

An individual meta-analysis was carried out for each group of patients (i) FTD alone, (ii) ALS alone and (iii) FTD and ALS patients combined as an overall evaluation of FTD-ALS spectrum disorders. Additional subgroup analyses included: an assessment of heterogeneity and a quality score. Outcome measures were calculated for each of the studies identified and included on a forest plot. Given the variability of intervention quantification included in the analysis, outcome measures were recorded in standardised mean differences (SMD), to allow for meaningful comparisons between studies. SMD was compared using Hedges g-statistic, to account for bias from small sample sizes, using a random effects model. SMDs were reported as odds ratios with 95% confidence intervals. Heterogeneity was assessed for all outcome measures using I^2^-values, and a funnel plot and Egger’s regression test was used to assess publication bias.

## Results

### Systematic review reveals poor reporting of measures to reduce bias but no evidence of publication bias

**Fig. 1:**
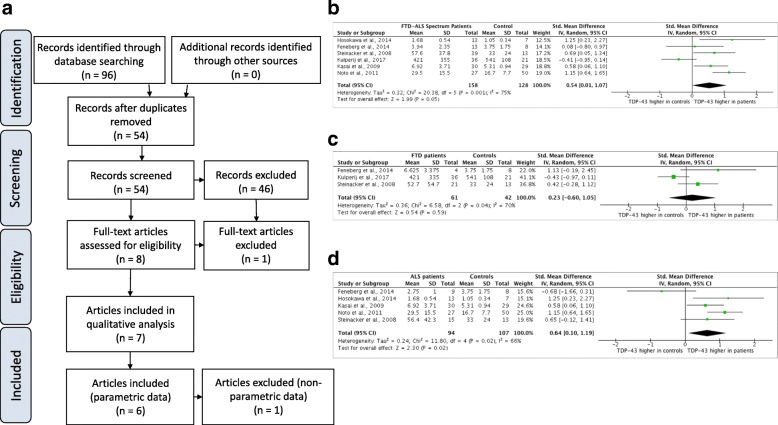
**a** PRISMA Diagram detailing each step of the systematic review, with number of studies highlighted as n = x. Reasons for exclusion given in boxes on the right. **b** Forest plot assessing the utility of CSF TDP-43 as a biomarker for FTD-ALS displaying standardised mean differences (SMD) and 95% confidence intervals (CI) using a random effects model. Study identifiers (author and year of publication) given on left. Heterogeneity calculated using Chi^2^ and I^2^ showing significant heterogeneity. Summary statistic (black diamond shows statistically significantly higher TDP-43 in the CSF of FTD-ALS patients compared to controls. **c** Forest plot assessing the utility of CSF TDP-43 as a biomarker for FTD displaying SMD and 95% CI using a random effects model. Study identifiers (author and year of publication) given on left. Heterogeneity calculated using Chi^2^ and I^2^ showing significant heterogeneity. Summary statistic (black diamond shows no statistically significant difference in TDP-43 in the CSF of FTD patients compared to controls. **d** Forest plot assessing the utility of CSF TDP-43 as a biomarker for FTD-ALS displaying SMD and 95% CI using a random effects model. Study identifiers (author and year of publication) given on left. Heterogeneity calculated using Chi^2^ and I^2^ showing significant heterogeneity. Summary statistic (black diamond shows statistically significantly higher TDP-43 in the CSF of ALS patients compared to controls

Overall, 6 studies were identified for meta-analysis, including a total of 274 participants [14–19], 150 neurological controls (Table 1) and 146 patients with FTD-ALS spectrum disorders (94 with ALS and 77 with FTD) (Fig. 1a). Seven studies were included in the qualitative analysis (Table 1; Fig. 1a). The data from the Suarez-Calvet et al., 2014 study [20] was non-parametric, so could not be compared to the other studies in the quantitative analysis. There was no evidence of publication bias detected by Egger’s regression (best-fit gradient of − 0.01017 ± 0.03182 and *p* = 0.8029) or funnel plot. Systematic review, risk of bias and quality assessment revealed that only 3 of the 7 identified papers reported on measures to reduce bias, such as blinding (Table 1). FIve of the studies used an ELISA technique to evaluate the concentration of TDP-43 in CSF samples [14–18] with the remaining two papers using a semi-quantitative analysis of immunoblot intensity. Suarez-Calvet et al., 2014 report concentrations as an ELISA comparison, with a manufactured TDP-43 standard. Steinacker et al., 2008 used an immunoblot intensity comparison between samples and a stock sample of CSF TDP-43 from the post mortem frontal lobe of a single FTD patient, which increases the risk of bias due to the semi-quantitative nature of immunoblotting. Four of the 7 studies were assessed as having a low risk of bias as determined by QUADAS-2 quality assessment (Table 1; [14–16, 19]) and 2 studies were assessed to have a risk of bias due to lack of blinding [17, 18]. The control populations used were different between studies, 2 studies used healthy controls [19, 20] and 4 used non-neurodegenerative neurological patients as controls [14, 16–18] with conditions such as focal complex seizures or depression. Kasai et al., used a combination of non-neurodegenerative neurological patients and healthy controls. All studies used an appropriate clinical diagnosis to confirm the diagnosis of ALS or FTD. Two studies used additional, supportive diagnostic modalities such as fMRI or post mortem lab testing [14, 18].

**Table 1:** Study characteristics

Study	Number of FTD patients	Number of ALS patients	Number of FTD and/or ALS patients	Number of controls	Males	Females	Age FTD (years)	Age ALS (years)	Age ALS-FTD (years)	Age control (years)	Pathology in controls	Screening technique	Analysis technique	Risk of bias
Hosokawa et al., 2014 [14]	N/A	13	13	7	12	8		59	59	49	GBS	Clinical diagnosis	ELISA	Low
Noto et al., 2011 [15]	N/A	27	27	50	N/A	N/A	N/A	N/A	N/A	N/A	PD, PSP, MS, MSA, GBS, FS	El Escorial and Awaji	ELISA	Low
Kasai et al., 2009 [16]	N/A	30	30	29	19	11	N/A	65	65	69	Nonneurodegenerative/neurological patients and healthy controls	El Escorial	ELISA	Low
Feneberg et al., 2014 [17]	4	9	13	8	14	7	66	64	65	62	Nonneurodegenerative/neurological patients	El Escorial	Western blotting and mass spectrometry	Semiquantitative analysis
Suarez-Calvet et al., 2014 [20]	25	N/A	25	22	29	18	67.8	N/A	67.8	70.1	Healthy controls	FTD consensus clinical criteria + CSF biomarker profile to exclude AD.	ELISA compared to manufactured control	No blinding
Kuiperji et al., 2017 [19]	36	N/A	36	21	N/A	N/A	N/A	N/A	N/A	N/A	Healthy controls	El Escorial	ELISA	Low
Steinacker et al., 2008 [18]	24	15	27	13	24	18	68	55	63	60	Nonneurodegenerative/neurological patients	El Escorial, DSMIV, fMRI, PM lab confirmation	Immunoblot intensity against internal standard	Semiquantitative analysis and concentration compared to single FTD sample
Total	77	94	171	150	98	62	Median = 67.8	Median = 61.5	Median = 65	Median = 65.5	N/A	N/A	N/A	N/A

Total sample sizes given at the bottom of the first four columns (Excluding Saurez-Calvet et al., [20]). Mean ages given at the bottom of the age columns. Where no further disambiguation was given for the ‘pathology in controls’, ‘Non-neurodegenerative neurological patients’ is stated. Diagnostic tools and further patient screening techniques are presented in the screening techniques column. Method of analysing CSF TDP-43 is stated in the ‘Analysis Techniques’ column. Risks of bias uncovered through quality screening using QUADAS-2 is given in the ‘Risk of Bias’ column. QUADAS-2 findings detailed in Appendix ii NA = Not Available. (*ALS* amyotrophic lateral sclerosis, *FTD* frontotemporal dementia, *GBS* Guillain-Barré syndrome, *PD* Parkinson’s disease, *PSP* progressive supranuclear palsy, *MS* multiple sclerosis, *MSA* multi-system atrophy, *FS* functional signs, *PM* post-mortem)

### Meta-analysis reveals a statistically significant increase in TDP-43 in the CSF of FTD-ALS and ALS patients, but not FTD patients alone

We conducted three separate meta-analyses of the included studies to evaluate the potential of CSF TDP-43 as a biomarker in each of (i) FTD-ALS spectrum disorders (ALS and FTD combined), (ii) FTD alone, and (iii) ALS alone. Meta-analysis of FTD-ALS spectrum disorders versus controls using standard mean difference found an effect estimate of 0.55 (95% CI: 0.01–1.09) and a Z-score of 2.0 (*p* < 0.05) demonstrating a significant increase in detectable TDP-43 in the CSF of patients with FTD or ALS compared to controls. However, Chi^2^ was 20.57 and I^2^ was 76% showing significant heterogeneity in this dataset (Fig. 1b). Meta-analysis of studies assessing TDP-43 in the CSF of patients with FTD alone showed an effect estimate of 0.50 (95% CI: -0.65 - 1.65; Fig. 1c), although this favours increased levels of TDP-43 in the control group, this finding does not reach statistical significance with a Z-score of 0.85 (*p* = 0.40). There was a similarly significant heterogeneity with a Chi^2^ of 10.75 and an I^2^ of 81%. Meta-analysis of studies assessing TDP-43 in the CSF of patients with ALS alone showed an effect estimate of 0.64 (95% CI: 0.10–1.19). A Z-score of 2.3 (*p* = 0.02) showing a significant increase in detectable TDP-43 in the CSF of ALS patients. Chi^2^ was 11.80 and I^2^ was 66% showing moderate heterogeneity (Fig. 1d).

## Discussion

To our knowledge, this is the first systematic review and meta-analysis of the literature assessing the utility of TDP-43 in the CSF of patients with FTD-ALS spectrum disorders as a potential diagnostic biomarker. We have shown that CSF TDP-43 is significantly increased in patients with FTD-ALS spectrum disorder. However, when analysing patients with ALS and FTD separately, only patients with ALS (not patients with FTD) showed a significantly increased CSF TDP-43. We also detected substantial heterogeneity and relative risk of bias in these studies. This heterogeneity is likely due to the wide variety of methods used to analyse CSF TDP-43 concentrations, but could also reflect the disease heterogeneity (both clinically and genetically) seen in these spectrum disorders and the heterogeneity of controls used for comparison. Two of the studies used normal healthy controls [19, 20] whilst the remaining included patients with a wide variety of neurological diagnoses as controls. Kuiperij et al. excluded patients with non-FTD-ALS motor deficits, meaning their data could attenuate the effect size of FTD-ALS spectrum and pure FTD patients in this meta-analysis, therefore increasing heterogeneity. Steinacker et al. compared their concentration of TDP-43 to a standard developed from a single patient with FTD. There is no indication of how representative this patient is compared to other FTD-ALS spectrum disorder patients and depending on the concentration of TDP-43 in their standard, this could affect the final reported TDP-43 concentrations. Risk of bias could also have had an impact on the heterogeneity seen. For example, neither Feneberg et al. nor Suarez-Calvet et al. commented on blinding in their methods, an important aspect of scientific rigor to ensure that all samples are treated and assessed equally, limiting the potential for bias. Furthermore, variation in time from sampling to storage, from 30 min to several hours (and in 5 out of 7 studies, this time was not documented), post CSF sampling may influence TDP-43 quantification due to protein degradation. Diagnostic criteria for patient identification did not vary between studies, all but two studies used the El Escorial criteria for ALS and all studies studying FTD used Consensus Criteria for FTD, and many of the studies went to further investigate a diagnosis of FTD, ALS or FTD-ALS by with more detailed imaging studies or post mortem [18].

Given the significant effect size shown in this meta-analysis, demonstrating an increase in the levels of detectable TDP-43 in patients with ALS, further research to refine the use of CSF TDP-43 as a diagnostic tool is warranted. Furthermore, a longitudinal study of CSF TDP-43 concentrations in patients with FTD-ALS spectrum disorders might show if CSF TDP-43 increases with disease progression. Similarly, a longitudinal study investigating carriers of the *C9orf72* hexanucleotide expansion might provide an indication as to how early TDP-43 aggregation occurs, and potentially would allow us to diagnose FTD-ALS spectrum disorders pre-symptomatically in these patients [4, 7, 21]. This would facilitate study of preventative and disease modifying interventions in early disease. However, given the wide range of techniques used to evaluate the concentration of TDP-43 in FTD-ALS spectrum disorder patients there clearly needs to be agreement with regards to consistency of sampling, storage and testing techniques, with a standardized operating protocol adhered to by all diagnostic laboratories. Ideally, a larger, much more highly powered study into the effect of CSF TDP-43 as a diagnostic tool for FTD-ALS spectrum disorders would be done. This study should have aged-matched controls and ALS, FTD and crucially FTD-ALS patients (patients with both ALS and FTD symptoms), with the controls having no neurological or psychiatric morbidity and the patients having no confounding co-morbidities or genetic variations (such as *C9orf72*) that might skew data. The study should be carried out longitudinally in order to map the progression of TDP-43 in both controls and patients. Diagnosis would be done using currently approved diagnostic tools, with post-mortem pathological verification of diagnosis. Both the sampler and the pathologist should be blinded to whether the CSF is from a control or a patient. Equally, analysis should be done with an ELISA and presented in empirical concentrations to improve transparency. As of yet, there are not enough studies into the TDP-43 in the blood, either in lymphocytes or in plasma. However, these are avenues for future meta-analyses (given more studies), as they provide less invasive diagnostic tests than lumbar puncture.

### Study limitations

Our data demonstrate a significant difference in TDP-43 levels in ALS patients compared to controls, implying that CSF TDP-43 levels show promise as a potential biomarker in ALS. It is probable that the statistically significant result observed in FTD-ALS spectrum disorder cases (Fig. 1b), could be due to the ALS patients included therein as FTD patients alone did not show a statistically significant increase in CSF TDP-43. This is likely reflected in the substantial heterogeneity seen in the combined analysis. However, whilst the lower confidence interval for FTD alone cases does intersect the line of no effect, the effect size and upper confidence interval are still favouring a higher level of TDP-43 in cases compared to controls, indicating that further studies are warranted to reduce the confidence intervals sufficiently to ascertain the potential of TDP-43 as a biomarker in FTD. Furthermore, whilst TDP-43 pathology is a unifying feature of the majority of ALS and FTD cases, TDP-43 accumulation is also observed in other neurodegenerative conditions; including but not limited to AD. Whilst our findings suggest that CSF TDP-43 may be a promising biomarker in ALS, it’s potential in the context of other neurodegenerative conditions is not yet fully understood. There are currently only two publications assessing the utility of TDP-43 as a biomarker in AD [22, 23], which we considered to be too small a sample to conduct a meta-analysis on. However, future systematic reviews should consider including an assessment of TDP-43 in other neurodegenerative diseases when these data become available.

## Conclusion

Our systematic review and meta-analysis reveals early supportive data indicating that TDP-43 detected in the CSF of patients with FTD-ALS spectrum disorders in particular ALS patients could be a promising biomarker in these diseases. Given the current paucity of diagnostic and prognostic biomarkers in these disorders, this result clearly indicates that further studies in to its utility are warranted.

## Abbreviations

AD: Alzheimer’s disease
ALS: Amyotrophic lateral sclerosis
CSF: Cerebrospinal fluid
DMT: Disease modifying treatments
ELISA: Enzyme-linked immunosorbent assay
FTD: Fronto-temporal dementia
TDP-43: TAR-DNA binding protein 43 kDa
